# Transduction of human dendritic cells with adenovirus encoding anti-PD-1 reduces PD-1 expression on co-cultured T cells

**DOI:** 10.1186/2051-1426-2-S3-P254

**Published:** 2014-11-06

**Authors:** Santos Patricia, Etheridge Alexander, Andrea Gambotto, Lisa H Butterfield

**Affiliations:** 1 Department of Medicine, University of Pittsburgh Cancer Institute, Pittsburgh, PA, USA; 2 SURP Immunology Program, Department of Immunology, School of Medicine, University of Pittsburgh, PA, USA; 3 Department of Surgery, School of Medicine, University of Pittsburgh, PA, USA; 4 University of Pittsburgh Cancer Institute, Pittsburgh, PA, USA

## 

A potent tumor-specific T cell response is an important part of antitumor immunity. Thus, enhancing T cell responses against tumor cells is a major focus in cancer immunotherapy. Dendritic cells (DC) play a critical role in the induction of T cell responses not only against pathogens but also against tumor cells. Studies have shown that DC-based vaccines are capable of presenting antigens via MHC Class I and MHC Class II molecules resulting in tumor antigen-specific T cell activation *in vitro *and *in vivo*. However, T cell responses against tumor antigens can be negatively regulated. For example, PD-1, which is up-regulated in activated T cells, can bind to PD-L1 or PD-L2 expressed in tumor cells as part of the immune suppressive tumor environment and thus inhibiting T cell activation. We wished to determine whether addition of anti-PD-1 to DC vaccines would result in enhanced tumor antigen-specific T cell responses. DCs transduced with recombinant adenovirus (AdV) encoding anti-PD1 (Ad5.hPD1Ab) secreted anti-PD1 in the supernatants which was able to bind to PD-1 expressed on the surface of HEK-293 cells via stable transfection. We examined the expression of surface markers associated with DC function and maturation 48 hours after transduction via flow cytometry. Our results show that Ad5.hPD1Ab transduced DCs had similar expression levels of antigen presentation molecules MHC Class I and II, costimulatory, and maturation related molecules CD40, CD80, CD83 and CD86 compared to DCs that were transduced with adenovirus encoding tumor antigens for hepatocellular carcinoma and melanoma (AdVhAFP and AdVTMM2, respectively). Furthermore, surface expression of inhibitory molecules PD-L1 and PD-L2 were also comparable among the three groups. Cytokine analysis show that 24-48 hours after transduction, Ad5.hPD1Ab transduced DCs secrete more IL-7, IL-15 and IP-10 than AdVhAFP and AdVTMM2 transduced DCs. Importantly, autologous T cells co-cultured with Ad5.hPD1Ab transduced DC results in reduced expression of not only surface PD-1 but also surface CTLA-4 inhibitory molecules in both CD4^+ ^and CD8^+ ^T cells.

